# Obtaining and Investigation of the *β*-Cyclodextrin Inclusion Complex with Vitamin *D*_3_ Oil Solution

**DOI:** 10.1155/2020/6148939

**Published:** 2020-08-17

**Authors:** Ryszhan Bakirova, Altynbek Nukhuly, Ainara Iskineyeva, Serik Fazylov, Meiram Burkeyev, Ayaulym Mustafayeva, Yelena Minayeva, Akmaral Sarsenbekova

**Affiliations:** ^1^Karaganda Medical University, Non-Commercial Joint-Stock Company, Karaganda, Kazakhstan; ^2^Pavlodar State Pedagogical University, Pavlodar, Kazakhstan; ^3^Saken Seifullin Kazakh Agrotechnical University, Nur-Sultan, Kazakhstan; ^4^Karaganda State University, Karaganda, Kazakhstan

## Abstract

*Background*. The research results of fat-soluble vitamin *D*_3_ (cholecalciferol) encapsulation with *β*-cyclodextrin have been presented in this work. The vitamin *D*_3_ inclusion complex with *β*-cyclodextrin was obtained under microwave radiation. The surface morphology of obtained clathrate inclusion complexes was described with the help of a scanning electron microscope. The thermographic measurement results on a differential scanning calorimeter have been presented. The activation energy of the *β*-cyclodextrin : vitamin *D*_3_ clathrate complex thermal oxidative destruction reaction was calculated. The clathrate thermal destruction kinetic parameters were determined. The inclusion complex spectral properties were characterized by IR-Fourier and ^1^H and ^13^C NMR spectroscopy. The existence of *β*-cyclodextrin inclusion complex with vitamin *D*_3_ in a 2 : 1 ratio was confirmed by the experimental results. The activation energy of thermal destruction of the inclusion complex of *β*-cyclodextrin with vitamin *D*_3_ was calculated using four different methods.

## 1. Introduction

**Figure 1:**
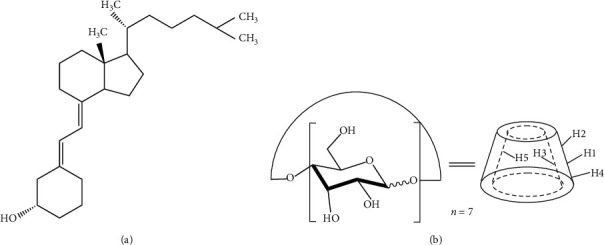
Structural formulas of cholecalciferol (a) and *β*-cyclodextrin (b).

Today, as per the latest medical reports available, majority of the population throughout globe is facing vitamin *D* deficiency. Vitamin *D* deficiency is now recognized as a pandemic [1, 2]. Vitamin *D*, also known as cholecalciferol, including vitamin *D*_2_ (ergocalciferol) and vitamin *D*_3_ (cholecalciferol), whose chemical name is 9,10-open-loop cholesteric-5,7,10(19-)leukotriene-3*β*-alcohol, and vasoactive substance is 25-hydroxy vitamin D_3_, abbreviated as [25-(OH)-D_3_] (calcifediol, INN) (Figure 1). In recent years, the demand for VD_3_ is on the rise, which is widely used in areas of food additives, pharmaceutical preparations, and feed additives. Vitamin *D*_3_ (VD_3_) is involved in calcium and phosphorus metabolism in a human body. This compound is necessary for the formation and maintenance of bones health, endocrine, and other human body systems. The recent research has further elaborated the role of VD_3_ in prevention of cancer, cardiovascular diseases, diabetes, cellular growth, cellular differentiation, embryonic development, fertility, immunological disorder, liver disorder, and neurological, renal, and respiratory disorders [2–7]. Millions of preschool-aged children are found to be VD_3_ deficient [2]. Food does not fully cover the needs for VD_3_. There is a need for additional food enrichment with vitamin in these cases. A large proportion of VD_3_ is lost during food processing and storage due to environmental stress conditions such as temperature, pH, salt, oxygen, and light.

In addition, lipophilicity and insolubility of VD_3_ in water (less than 1 mg/100 g) create difficulties for its application in technological processes. To use fat-soluble vitamins and antioxidants as food additives in dairy and other agricultural products, you need to get their water-soluble form. The water-soluble form will improve the bioavailability and effectiveness of vitamins. Recent advances in nanotechnology offer various microencapsulation techniques such as liposome, solid-lipid particles, nanostructured lipid carriers, emulsion, and spray drying, which have been used to design efficient nanomaterials with desired functionality and have great potential for enrichment of fortificants like VD_3_ [2]. The complexation of vitamins with cyclodextrins also eliminates these disadvantages; therefore, fundamental research in this area is of great theoretical and applied importance [8–11].

Cyclodextrins (CDs) are cyclic oligosaccharides that have an inner hydrophobic cavity and a hydrophilic outer shell (Figure 1). They are starch biochemical transformation products. The CD family includes three main products, namely, *α*-CD, *β*-CD, and *γ*-CD, macrorings of which consist of six, seven, and eight glucopyranose residues, respectively. Hydrophobic molecules are able to integrate into the CD internal cavity, forming “guest-host” type inclusion complexes [12–14].

This provides significant changes in the physicochemical properties of the molecules bound by the cyclodextrins of the substance: the stability of the “guest” substances sensitive to the effects of oxygen or light increases [15–18], the solubility of the substances increases [11, 19, 20], and the possibility of converting liquids into powder form [14, 15, 21], unpleasant odors, and taste are masked [8, 18, 22]. X-ray diffraction, thermoanalytical, and mass spectrometric measurements confirmed that VD_3_ (cholecalciferol) forms an inclusion complex with *β*-cyclodextrin. This complex formation (molecular encapsulation) improves water solubility [23]. C NMR spectra of the complex of VD_3_ with heptakis-(2,6-di-O-methyl)-*β*-cyclodextrin prove that two rings of cyclodextrin form a capsule around one molecule of VD_3_ [16]. In [24], trace amounts of VD_3_ were wrapped in a *β*-CD molecule by saturated aqueous vacuum drying. The process of obtaining the VD_3_ : *β*-CD complex was carried out with a stoichiometry of 1 : 15 and with stirring for 5 hours at 80°C.

*β*-CD сomplexation with a native cholecalciferol molecule can occur by incorporating a vitamin molecule's hydrophobic parts (nonpolar aliphatic or cyclic hydrocarbon radicals) into the *β*-CD cavity, while its hydrophilic parts (polar hydroxyl groups) are located outside the *β*-CD cavity [25–29]. The inclusion of cholecalciferol in *β*-CD leads to an increase in the thermal stability of the vitamin and its resistance to light, oxygen, and inorganic salts [23, 24]. The solubility of cholecalciferol in water as a complex with *β*-CD is 0.21 mg per 100 ml at 37°C. Vitamin *D*_3_ completely decomposes at 80°C for 24 hours, while its complex with *β*-cyclodextrin still retains 49% of its original activity even after 43 days [23]. The biological (antirachitic) activity of the inclusion compound of cholecalciferol with *β*-CD has been studied in rats fed with a vitamin *D*_3_-deficient diet. It was shown that free and *β*-CD-associated vitamins *D*_3_ have qualitatively similar effects; however, when using the inclusion compound, normalization of blood calcium and phosphorus levels and bone mineralization occur faster [23].

**Figure 2:**
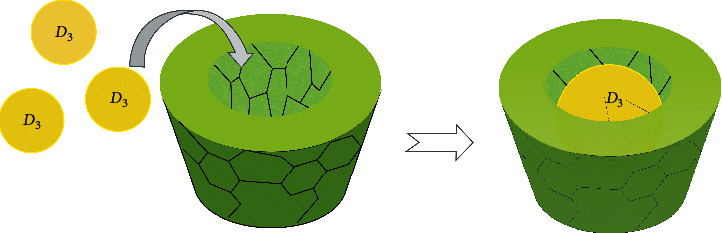
Schematic representation of the “guest-host” inclusion complex formation.

In production conditions, the lipophilicity and low solubility of the native form of VD_3_ in the water environment create certain difficulties and limit its use in the additive field. For this reason, there is a need to develop technological methods for obtaining water-soluble clathrate forms of vitamin with improved biopharmaceutical and nutritional properties. Therefore, it is important to fully understand the nature of the inclusion of VD_3_ in the complex “*β*-CD : VD_3_”. This form should also make it convenient to add VD_3_ to other foods (Figure 2). This paper presents the results of encapsulation of an oil (in olive oil) solution of VD_3_ (cholecalciferol) with *β*-cyclodextrin (*β*-CD). The application of a VD_3_ molecule oil solution should facilitate entry of vitamin into *β*-cyclodextrin molecules cylindrical hydrophobic cavities with a “guest-host” complex formation. In addition, VD_3_ will be better preserved in the oily shell from oxidizing agent effects and has better bioavailability. To obtain guest-host inclusion complexes, methods of codeposition, kneading, freeze-drying, as well as methods of ultrasonic and microwave technology are used [8, 25, 30]. According to recent data, carrying out inclusion reactions using microwave heating has the advantages of a shorter reaction time and higher product yield in contrast to conventional methods [25]. In the present work, we prepared the inclusion complex of *β*-CD : VD_3_ under microwave irradiation. The complex obtained was studied using FT-IR, SEM, DSC, ^1^H, and ^13^C NMR spectroscopy.

## 2. Materials and Methods

The following reagents were used, namely, *β*-cyclodextrin (99.5%, purchased from Fluka), vitamin *D*_3_ (cholecalciferol in olive oil (hereinafter vitamin *D*_3_)), “Healthy Origins”, 250 mcg (10.000 IU), cholecalciferol, С_27_Н_44_О, “analytical grade” (“Aldrich” company), white powdery substance. Mol. mass is 384.64 g/mol, mp. 84°C–85°C. The ^1^H NMR and ^13^C NMR measurements were carried out in DMSO-d_6_ (Aldrich) solutions, and other chemicals were of analytical reagent grade purity.

The surface morphology of *β*-CD : VD_3_ inclusion complexes (clathrates) samples was studied using a scanning electron microscope (SEM) from Tescan Mira 3 LMN (Czech Republic). IR spectra were recorded on a Cary 600 Series IR-Fourier spectrometer manufactured by Agilent Technologies (USA) in the range of 4000–400 cm^−1^. Samples were prepared from test compounds and KBr with a mass ratio of 1 : 100. The clathrates obtained ^1^H and ^13^C NMR spectra were recorded on a JNM-ECA Jeol 400 spectrometer (frequencies 399.78 and 100.53 MHz, respectively) using a DMSO-d_6_ solvent. Chemical shifts were measured relative to DMSO-d_6_ residual protons or carbon atoms signals. All measurements were performed at a resolution of 4.0 cm^−1^; the number of scans was 40. Microwave irradiation was carried out in a Galanz WP 700L20 microwave oven (Guangdong, China) at atmospheric pressure. The complex melting points were determined on a Boetius instrument (Germany). *β*-CD and inclusion complex with VD_3_ samples were analyzed by the thermographic method (the sample weight was equal to 12 mg). The thermal properties were studied on a Labsys Evaluation DTA/DTS differential scanning calorimeter in a dynamic mode at a temperature range of 30–500°C when heated at a rate of 10 deg/min in nitrogen atmosphere and in air in an Al_2_O_3_ crucible.

The *β*-CD inclusion complexes with VD_3_ were obtained according to the procedure [30]. A mixture of 0.4 mmol of *β*-CD and 0.2 mmol of VD_3_ was dissolved in ethanol : water (1 : 1) solvent mixture and microwaved for 180 s at 60°C. Under these conditions, the outputs of the inclusion complexes were 95–97%. Solvents were removed after the reaction, and the precipitate was washed with acetone and dried in a desiccator with CaCl_2_ to a constant weight. The resulting product is a white powder and soluble in water with colloidal solutions to form a milky white color. Cholecalciferol solubility in distilled water in a form of a complex with *β*-CD was 0.20 ± 0.05/100 ml.

## 3. Results and Discussion

**Figure 3:**
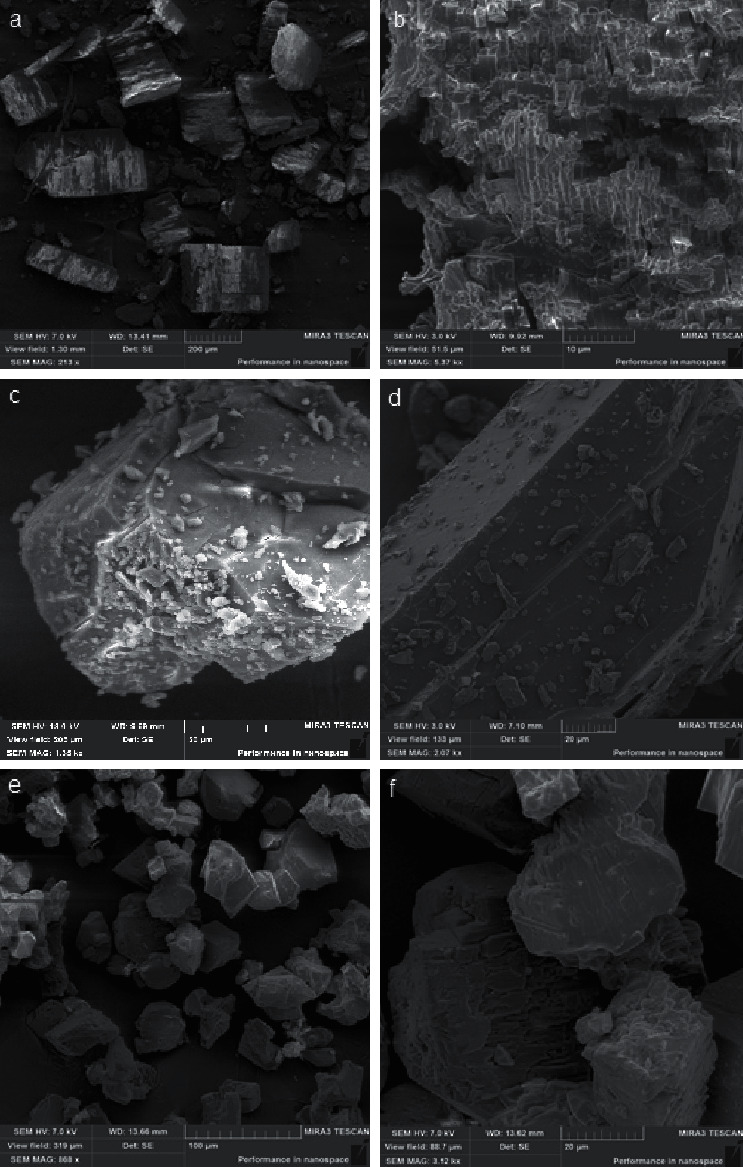
Scanned electron micrographs of *β*-cyclodextrin (a, b), a physical mixture of *β*-CD with VD_3_ (c, d), and *β*-CD : VD_3_ inclusion complex (2 : 1) (e, f) at various magnifications.

The *β*-CD particles and binary systems morphology were analyzed using SEM and presented in Figure 3. The SEM method is a qualitative method used to study objects under investigation structural aspects and helps to assess the presence of another component in the resulting preparations. Figure 3 shows the *β*-CD : VD_3_ inclusion complex (2 : 1) scanned electron micrographs. The clathrates samples studied were previously sprayed with a carbon conductive layer. Pictures were taken at an accelerating voltage of 3 and 7 kV. The *β*-cyclodextrin layered structure is visible in the samples in Figures 3(a) and 3(b) and a physical mixture of *β-*cyclodextrin with native cholecalciferol (VD_3_)-in Figures 3(c) and 3(d). A sharp change in the crystals morphology is observed in the Figures 3(e) and 3(f) of *β*-CD : VD_3_ samples (2 : 1). The crystalline forms are covered with a film. Similar results were reported in [31, 32]. Changes in crystal surface morphology are convincing evidence of the inclusion complex formation.

**Figure 4:**
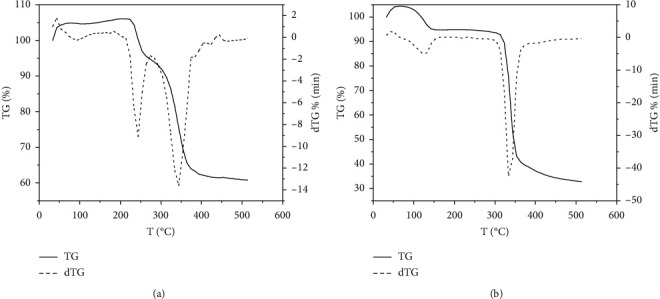
TG/DTG curves of the *β*-CD:VD_3_ with a constant heating rate of 10 deg/min in a nitrogen medium: (a) physical mixture; (b) *β*-CD:VD_3_*β*-CD:VD_3_ inclusion complex (2:1).

TG and DTG analyses of *β*-CD clathrates with VD_3_ were performed by differential thermogravimetry (DTG) and differential scanning calorimetry (DSC) using a Setaram differential scanning calorimeter DTA/DSC. Thermograms were taken under the following conditions, namely, Al_2_O_3_ crucible, nitrogen atmosphere, air, 30–800°С temperature range, samples heating rate from 5 to 20 K/min, and sample weights of 12–16 mg. All calculations were performed using the Mathcad program [33, 34]. The DSC method was used to identify the complexes based on a comparison of starting materials and synthesis product thermograms. Figures 4(a) and 4(b) present *β*-CD and VD_3_ physical mixture DSC thermograms, in which the endothermic peaks correspond to the compounds melting points. The peak at 84–85°С in the DSC thermogram corresponds to the VD_3_ melting point, and several peaks in the 240–348°С temperature range correspond to oxidative destruction processes of VD_3_, *β*-CD, and their decomposition products. Thermoanalytic indicators of *β*-CD : VD_3_ (2 : 1) decomposition are represented by TG/DTG curves (Figures 4(a) and 4(b)). Thermographic analysis data at various heating rates showed that *β*-CD and *β*-CD : VD_3_ clathrate differed in the temperature of thermal decomposition reaction onset and in the nature of the samples mass loss when heated up to 500°C.

**Figure 5:**
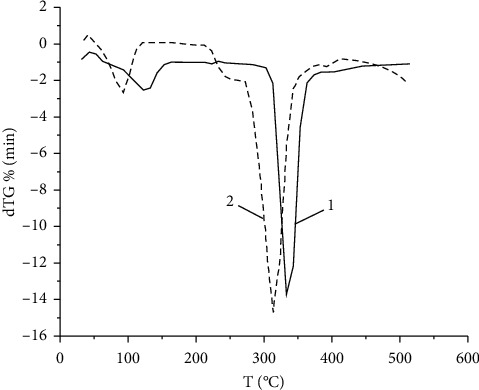
Differential thermographic curves (DTG) of *β*-CD (1) and *β*-CD : VD_3_ (2 : 1) (2) at a constant heating rate of 10 deg/min in a nitrogen medium.

The *β*-CD : VD_3_ clathrates obtained contained bound water as by *β*-CD. The samples dehydration endothermic peak was in the range of 70°C–100°C (Figure 4(a)). The “shoulder” appearance on the *β*-CD : VD_3_ DTG thermographic curve in the region of 210°C–240°C (Figure 4(b)) is most likely to be attributed to the VD_3_ thermal decomposition since its size increases with increasing VD_3_ concentration. The heat absorption peak caused by activation of thermal destruction is in the range of 270°C–320°C for *β*-CD : VD_3_ and 280°C–340°C for pure *β*-CD (Figure 5), which indicates a decrease in the cyclodextrin thermal stability when vitamin *D*_3_ is included in its internal cavity. It should be noted that the total mass loss at five heating rates was 74.9–81.6%. Changes in the relative mass at various heating rates are manifested at temperatures in the range of 200–450°С in all the dependences. Several zones of intense mass loss in the 50–100°С, 220–350°С, and 360–450°С temperature ranges can be determined on the differential curves (Figures 4 and 5).

The first zone corresponds to the water loss by the clathrate; the second one corresponds to the cyclodextrin ring destruction; the third one corresponds to the oxidation of products formed during the cyclodextrin ring destruction. A change in a heating rate of the samples under study did not affect the TG and DTG curves course, and no new peaks were detected. An increase in a heating rate leads only to an insignificant change in the temperatures of peaks beginning, the peaks minimum, and the end of the curve deviation from the baseline.

A comparative analysis of the *β*-CD and *β*-CD : VD_3_ thermograms shows that the *β*-CD : VD_3_ clathrate is characterized by a maximum of heat release at a temperature of 230°C–280°C. In this case, the *β*-CD thermal decomposition maximum shifts from 340°C to 320°C. These results also indicate the inclusion complexes formation. The activation energy of the *β*-CD : VD_3_ thermal oxidative degradation reaction was calculated by the *Freeman–Carroll* (a), *Sharp–Wentworth* (b), *Ahara* (c), and *Coats–Redfern* (d) methods [33, 34] (Table 1).

The activation energy is minimal (169.42 kJ/mol) at a lower VD_3_ concentration (*β*-CD : VD_3_, 2 : 1), and it begins to increase (229.12 kJ/mol) with VD_3_ concentration increase, which may indicate not only conformational changes in the *β*-cyclodextrin structure but also the *β*-CD : VD_3_ clathrate complex formation.

In the *β*-CD and *β*-CD : VD_3_ IR spectra, O-H bond stretching vibrations are found in the form of a wide band with a maximum at 3387 cm^−1^ in all binary systems. There is also an absorption band at 2924 cm^−1^ characteristic for stretching vibrations of CH bonds in the CH and CH_2_ groups. An absorption band at 1651 cm^−1^ is characteristic for deformation vibrations of the OH bond in the СОН groups, and absorption bands at 1423, 1364, and 1335 cm^−1^ are due to deformation vibrations of the С-H bonds in the CH_2_OH and CHOH groups [23, 25, 28]. Absorption bands of C=C, OH hydroxyl bonds, and other cholecalciferol groups do not appear in the IR spectra of the *β*-CD : VD_3_ complex. This may mean that these groups are masked by very wide and intense *β*-CD bands in the same wavelength range.

One of the informative methods for confirming the inclusion complexes formation is the ^1^H NMR spectroscopy method [22, 35–37]. The *β*-CD molecule has a truncated cone shape, in the inner hydrophobic binding surface of which H-3 and H-5 protons are located, and H-2 and H-4 protons are on the outer one [18, 21, 27]. This analysis method allows fixing a pronounced chemical shift in the *β*-CD H-3 and H-5 protons vibrational spectra oriented inside a torus cavity, which is due to the guest molecule placement in the cyclodextrin hydrophobic cavity.

According to our studies [38, 39], the six groups of signals manifestation in the region of 3.32–3.35, 3.45–3.65, 4.48–4.55, 4.78–4.82, and 5.67–5.76 ppm is characteristic for the individual *β*-CD ^1^H NMR spectrum. The most low-field doublet signal in the range of 5.71–5.73 ppm with a splitting of 4 Hz belongs to the hydroxyl group proton at the C-2 atom. The OH group proton of a neighboring atom (OH-3) located in the *β*-CD molecule internal cavity also resonates in the weak field. Doublet signal in the region of 4.78–4.82 ppm corresponds to the H-1 proton. The location of this proton in a weaker field compared to the protons of other CH groups is due to the oxygen atom influence. H-6a, *b* signals of the methylene group are observed in the region of a strong field (3.58–3.65 ppm). High-intensity signal at 3.46 ppm corresponds to the H-3 proton of a glucopyranose link. ^13^C nuclei six groups of signals of the initial *β*-CD elementary unit are also presented in Table 2. The C-6 atom signal appears at 60.41 ppm. Signals at 72.49, 72.85, and 73.51 ppm are caused by C-5, C-2, and C-3 atoms, respectively. C4 and C-1 carbon atoms signals are observed in the region of 82.02 and 102.41 ppm, which are directly connected to the adjacent glucopyranose link through the oxygen bridge.

The ^1^H and ^13^C NMR chemical shift values of *β*-CD in a free and complexing state are shown in Table 2. All six *β*-CD protons show a pronounced chemical shift towards a strong field. The largest difference in the chemical shift values Δ*δ* in the *β*-CD : VD_3_^1^H NMR spectrum is characteristic for the H-3 and H-5 intraspheric protons. However, it can be concluded that an internal (inclusive) complex is formed in a clathrate [21, 33, 38–44]. In the case of the carbon spectrum, there is a more significant difference in the change in chemical shifts, which ranges from 0.06 to 0.22 ppm. A proportional increase in the chemical shift in the ^1^H and ^13^C NMR vibrational spectra is observed with an increase in the “guest” compound (VD_3_) concentration in the system due to the equilibrium state shift towards the inclusion complex formation. These observations prove the reality of inclusion and show that hydrophobic interactions are driving forces for the inclusion complex formation [30, 35–37, 40].

## 4. Сonclusions

The *β*-cyclodextrin with vitamin *D*_3_ encapsulated inclusion complex was obtained. The inclusion complex was obtained under the influence of microwave radiation with the target product outputs 93–95%. The preparation of the clathrate complex *β*-CD : VD_3_ led to a change in the state of aggregation of the oil solution of vitamin *D*_3_, as well as an increase in its solubility in the aqueous medium. Cholecalciferol solubility in distilled water in a form of a complex with *β*-CD was 0.20 ± 0.05/100 ml. The *β*-CD : VD_3_ complex synthesized refers to “host-guest” inclusion compounds. In this case, the “guest” compound molecules enter into an encapsulated state, being located in the cyclodextrin internal cavity. SEM, TG, and DTG as well as ^1^H and ^13^C NMR spectroscopy data of *β*-cyclodextrin clathrate with vitamin *D*_3_ indicate its formation. Thermographic analysis data at various heating rates showed that *β*-CD and *β*-CD : VD_3_ clathrates differ in the temperature of the onset of the thermal decomposition reaction and in the nature of the mass loss of the samples when heated to 500°C. Mathematical processing of kinetic data was performed, describing the kinetics of the process using kinetic models known in the literature. The decisive role in the clathrate complex formation belongs to nonspecific (hydrophobic, dispersion, and van der Waals) interactions. A proportional increase in the chemical shift in the ^1^H NMR vibrational spectra is observed with an increase in the guest compound (vitamin *D*_3_) concentration in the system due to the equilibrium state shift towards the inclusion complex formation.

## Figures and Tables

**Table 1:** *β*-CD : VD_3_ (2 : 1) thermal degradation kinetic parameters in a nitrogen medium.

Sample	Freeman–Carroll method		Sharp–Wentworth method		Ahara method		Coats–Redfern method	
	E (kJ/mol)	*n*	E (kJ/mol)	A × 10^14^ min^−1^	E (kJ/mol)	A × 10^11^ min^−1^	E (kJ/mol)	A × 10^8^ min^−1^
*β*-CD : VD_3_	271.34	5.93	359.75	2.33	289.53	3.62	282.66	1.00

**Table 2:** ^1^H and ^13^C NMR chemical shifts in a free state and as a part of the *β*-CD : VD_3_ (2 : 1) inclusion complex.

Atom number	CH_X_ group	Β-CD (*δ*_0_) (ppm)		Β-CDv : VD_3_ (*δ*) (ppm)		Δ*δ* (*δ*-*δ*_0_) (ppm)	
		*δ* (^1^Н)	Δ (^13^С)	*δ* (^1^Н)	Δ ^(13^С)	*δ* (^1^Н)	Δ ^(13^C)
1	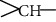	4.829	102.43	4.727	102.21	−0.102	−0.22
2	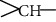	3.579	72.87	3.473	72.81	−0.106	−0.06
3	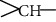	3.516	73.54	3.404	73.39	−0.112	−0.15
4	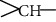	3.472	82.13	3.357	82.00	−0.015	−0.13
5	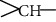	3.357	72.51	3.349	72.39	−0.108	−0.12
6	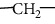	3.629	60.40	3.550	60.30	−0.079	−0.10

## Data Availability

The data used to support the findings of this study are available on request to the corresponding author.
